# Transradial Retrograde Percutaneous Coronary Intervention of Chronic Total Occlusion *via* an Ipsilateral Septal Collateral Using a Single Guiding Catheter: A Case Report

**DOI:** 10.3389/fcvm.2022.814492

**Published:** 2022-02-09

**Authors:** Xiaogang Liu, Jing Zhang, Hong Zhang, Peng Zhang, Naikuan Fu

**Affiliations:** Department of Cardiology, Tianjin Chest Hospital, Tianjin, China

**Keywords:** chronic total occlusion, percutaneous coronary intervention, retrograde approach, radial artery access, case report

## Abstract

**Background:**

With the development of specialized equipment and the retrograde technique, success rates for percutaneous coronary intervention (PCI) of chronic total occlusions (CTOs) have increased from 60 to 90% in the past 10 years. Performing PCI *via* a collateral channel from the contralateral artery, using two guiding catheters, is usually the preferred approach to retrograde CTO-PCI. In the case described in this report, only the ipsilateral septal collateral artery from the proximal occluded left anterior descending (LAD) artery was available. The procedure can be performed successfully from radial artery access using a single guiding catheter.

**Case Summary:**

A 57-year-old patient, with a history of anterior and inferior myocardial infarction and previous PCI, underwent a planned coronary arteriography due to his complaints of typical angina symptoms. Coronary angiography revealed stent occlusion located mid-LAD and severe in-stent restenosis in the distal right coronary artery (RCA). A proximal septal branch was supplying the distal LAD retrogradely. After repeated failed attempts at antegrade PCI for the LAD's CTO, the retrograde approach was tried. This intervention finally succeeded through the ipsilateral septal collateral. It was performed *via* a single radial-artery access throughout the whole process. Post-operatively, the patient had no complications and was stable at 1-year follow-up.

**Conclusion:**

The transradial approach to retrograde PCI for CTO *via* an ipsilateral septal collateral using a single guiding catheter is feasible and safe in appropriately selected cases.

## Introduction

Successful percutaneous coronary intervention (PCI) of chronic total occlusions (CTOs) has been associated with a reduced need for coronary artery bypass graft surgery (CABG), improvements in left ventricular function, and better long-term survival (1). With the development of specialized equipment and the retrograde technique, success rates for recanalization of CTOs have increased from 60 to 90% in the past 10 years (2, 3). The advantages of the retrograde approach in CTO-PCI have been observed in many clinical cases. Using septal collaterals from the contralateral artery as an access route shows a high success rate in retrograde CTO-PCI. However, not all CTO cases are well-suited for using the contralateral septal collateral as an access route. In some cases of left anterior descending (LAD) lesions, only the ipsilateral septal collateral from the same artery is observed to supply the distal recipient artery (4).

Femoral arterial access is usually the preferred approach to CTO-PCI because of its ability to firmly support a larger guiding catheter (7-French), but in several recent reports, radial access has been demonstrated to be possible, safe, and effective (5). We present a case of mid-LAD CTO recanalization performed successfully *via* an ipsilateral septal collateral, using a single guiding catheter that was positioned in the radial artery.

## Case Presentation

A 57-year-old patient was admitted to the cardiovascular department with progressive angina and dyspnea over 6 months. The patient's past medical history included anterior and inferior myocardial infarction, which was treated with PCI of the anterior descending branch and right coronary artery (RCA) 17 years prior, and hypertension. He was on several medications for 3 months, including clopidogrel, aspirin, atorvastatin, metoprolol, telmisartan, furosemide, and spironolactone. The physical examination was notable for rales at the lung bases and mild lower-extremity edema. The remainder of the physical examination was unremarkable.

**Figure 1:**
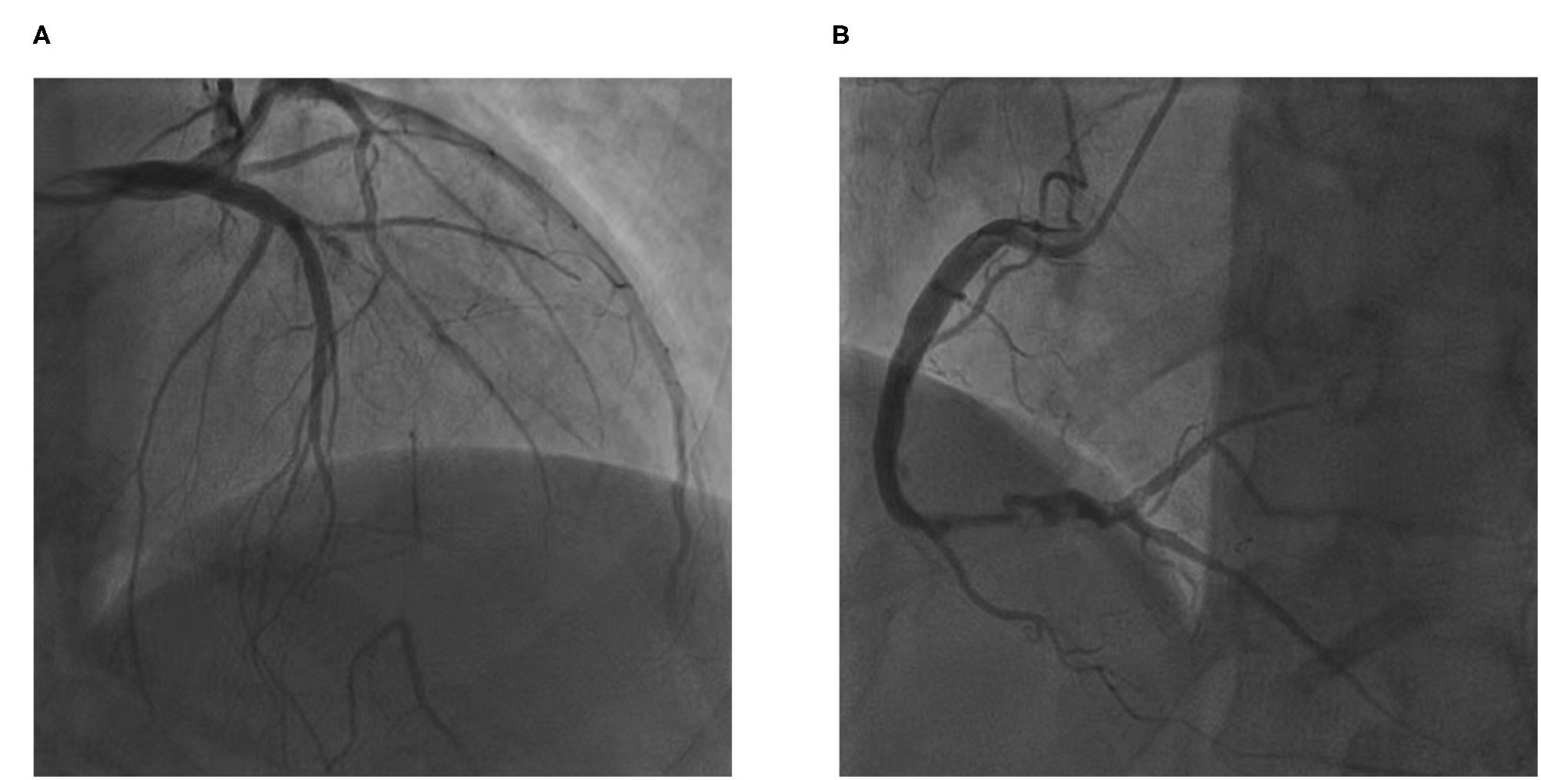
The patient's coronary angiography. **(A)** In-stent chronic total occlusion of left anterior descending artery and a proximal septal branch supplying the distal left anterior descending (LAD). **(B)** Severe in-stent restenosis of right coronary artery.

The electrocardiogram showed pathologic Q-waves in the anterior and inferior leads. Transthoracic echocardiography showed impaired left ventricular function (EF = 38%) and a dilated left ventricle (LV = 75 mm). The patient was diagnosed with coronary heart disease with unstable angina and heart failure. After receiving intensive anti-anginal and anti-heart failure medications, the patient underwent planned coronary arteriography a week later. Coronary angiography showed stent occlusion in the mid-LAD (Figure 1A) and severe in-stent restenosis in the distal RCA (Figure 1B). A proximal septal branch was supplying the distal LAD retrogradely (Figure 1A). The SYNTAX (Synergy between PCI with Taxus and Cardiac Surgery) score was 14.5.

**Figure 2:**
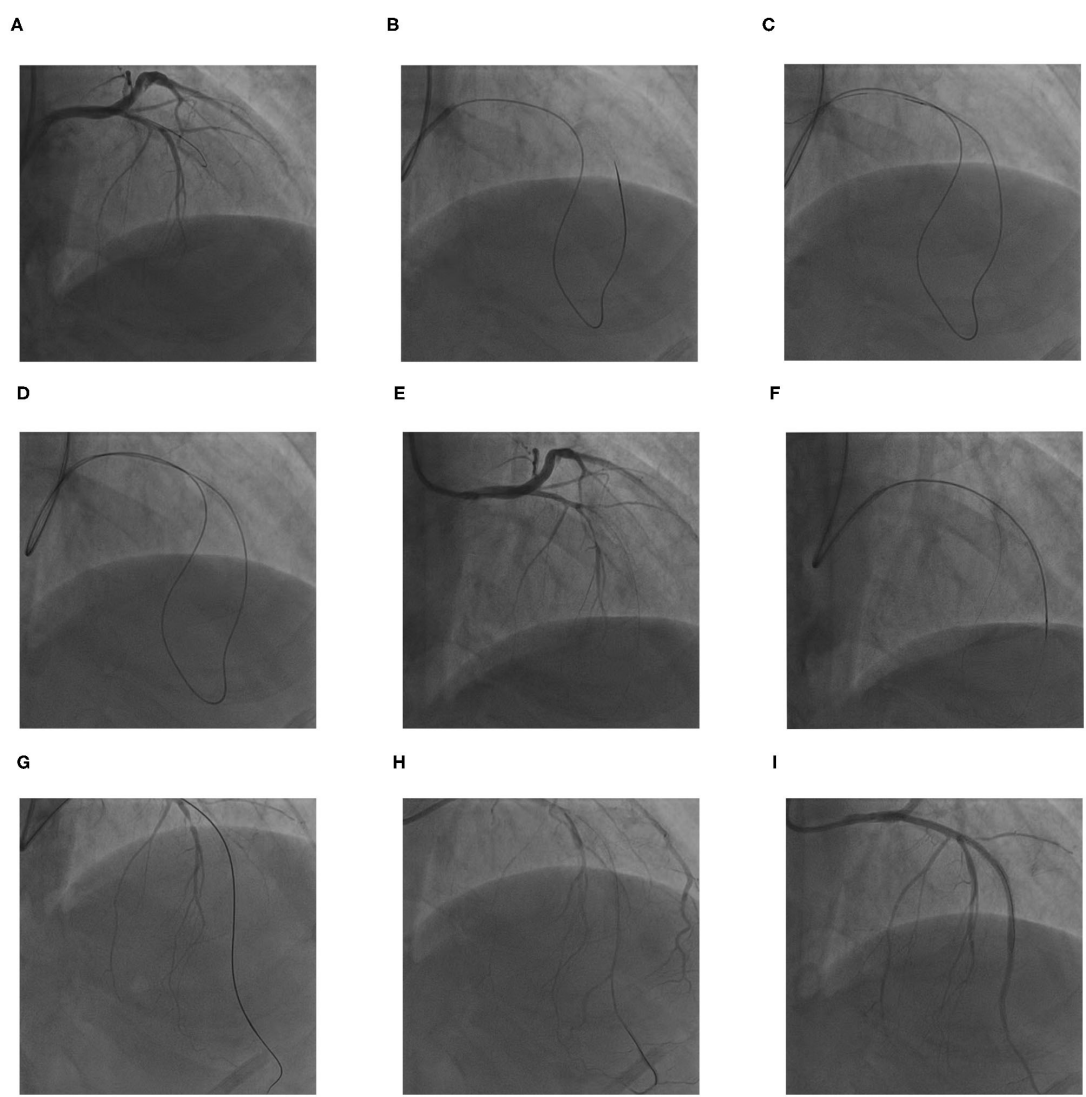
The procedures for retrograde percutaneous coronary intervention (PCI) of LAD chronic total occlusion (CTO). **(A)** The antegrade wire could not cross the CTO lesion. **(B)** The retrograde soft wire was advanced through the septal collateral with the microcatheter reaching the distal CTO lesion. **(C,D)** The retrograde soft wire was exchanged for a stiff wire *via* microcatheter, which crossed the CTO segment into proximal LAD true lumen, and threaded into the antegrade catheter with microcatheter retrogradely. **(E)** A 300-cm RG3 wire was externalized *via* the microcatheter. **(F)** The microcatheter was inserted into the proximal LAD through the septal collateral antegradely; the RG3 wire was removed. **(G,H)** The microcatheter was introduced to the distal LAD; the antegrade wire was advanced through the lesion. When the wire was confirmed in true lumen by angiogram, the microcatheter was removed. **(I)** Three drug-eluting stents were deployed at mid LAD successively.

Coronary artery bypass graft (CABG) surgery was advised for the patient according to the coronary angiography. The patient was thoroughly informed of the risks of the CTO-PCI; he refused CABG surgery and accepted the risks of PCI. PCI for the LAD CTO was then attempted. After repeated attempts at antegrade access failed (Figure 2A), the retrograde approach was tried. The 6-Fr JL 3.5 guiding catheter of radial artery continued to be used because the patient's radial spasm made changing sheaths difficult. In addition, there were concerns that his poor left-ventricular function would make lying flat over a long period risky; therefore, femoral access was the back-up site if transradial access failed. The retrograde wire (SION black 190, Asahi) was advanced through the septal collateral with the microcatheter (Corsair 150, Asahi), reaching the distal CTO lesion (Figure 2B). Then, the retrograde Sion wire was exchanged for a PILOT150 (Abbott) wire. It crossed the CTO segment successfully and was threaded into the guiding catheter retrogradely. The microcatheter was subsequently advanced over the wire into the guiding catheter (Figures 2C,D). A 300-cm RG3 wire (Asahi) was externalized *via* the microcatheter (Figure 2E). Then, the microcatheter was withdrawn retrogradely and advanced to the proximal LAD through the septal collateral over the wire antegradely. The RG3 wire was removed (Figure 2F). Then, the microcatheter was introduced to the distal LAD. Afterward, the antegrade wire (SION black) was advanced through the lesion. When the wire was confirmed to be in the true lumen by an angiogram, the microcatheter was removed (Figures 2G,H), and three drug-eluting stents were deployed successively at mid-LAD (Figure 2I). The final angiogram showed Thrombolysis in Myocardial Infarction (TIMI) flow grade 3 in the distal LAD.

After the intervention, the patient continued to be treated with guideline-directed medical therapy. The patient had no complications and underwent selective PCI of the RCA 2 weeks later. The patient was stable over 1 year of follow-up appointments. A detailed timeline of the events and therapy of the patient is provided (Table 1).

**Table 1:** Timeline.

**Timeline**	**Events**	**Coronary angiography**	**Intervention**
February 2002	Acute anterior and inferior myocardial infarction	1. Acute occlusion of left anterior descending artery (LAD) 2. Severe restenosis of right coronary artery (RCA)	Urgent percutaneous coronary intervention (PCI) of LAD and selective PCI of RCA
November 2019	Typical angina symptoms	1. In-stent chronic total occlusion (CTO) of LAD 2. Severe in-stent restenosis of RCA	Retrograde CTO PCI of LAD and selective PCI of RCA
December 2020	1-year follow-up: symptom free	Absence	Drug therapy

## Discussion

Chronic total occlusion (CTO) is defined as an occlusive lesion of over 3-month duration. The CTO-PCI tends to be a challenge for surgeons because of its low success rate and lengthy procedure. The main cause of CTO-PCI failure is usually an inability to cross the lesion (6). Improvements in the retrograde approach have helped increase the success rate. The proximal fibrous cap of a CTO lesion is characteristically hard and thick (7), which may increase the failure rate and the risk of subintimal dissection, while the distal cap is thinner and tapered so that the wire can cross it easily. This provides a theoretical basis for retrograde PCI. In the case described here, a large septal branch arising from the proximal LAD CTO was visible; otherwise, mid-LAD had a long occlusion. These observations suggested that retrograde PCI would be relatively easier to accomplish when antegrade attempts failed repeatedly.

Selecting a suitable collateral channel is the key to the success of retrograde approach. The selectable collateral channel for retrograde PCI could be a septal collateral or an epicardial collateral. Septal collaterals are preferred in majority of cases because, compared to the epicardial collaterals, they usually have a shorter and less tortuous course. Furthermore, they are less likely to cause tamponade when they are injured (8). In this case, no septal collateral from the RCA to the LAD was observed due to the severe stenosis in RCA. Instead, a septal collateral from the proximal LAD was found filling the distal LAD retrogradely, so it was chosen to be the retrograde access.

In most retrograde PCI cases, surgeons use the double-guiding catheter technique (9), but a single-guiding catheter was used *via* the radial artery in this case because of the patient's radial spasm and his poor left ventricular function. This reduced his time lying flat and minimized the risk of the onset of acute heart failure. The transradial approach could lower the risk of access-site complications, which include femoral artery hematoma and bleeding, and the risk of the onset of acute heart failure for patients with impaired heart function. Additionally, it may increase patient comfort and shorten hospital stays.

Nevertheless, this approach has limitations. First, it is the fact that smaller-size guiding catheters in the radial artery are less supportive and harder to operate. Second, when multiple devices need to be used simultaneously, a larger guiding catheter is more appropriate. Therefore, radial CTO PCI with a single-guiding catheter can be effective in appropriately selected cases. Third, if the retrograde wire has difficulty crossing the CTO segment or the microcatheter is difficult to advance across the CTO segment, the controlled antegrade and retrograde tracking (CART) technique, Knuckle technique, or anchor-balloon technique would be needed. In such cases, a second arterial access and a large-size guiding catheter would be required. Fourth, compared to the RG3 wire externalization, the “rendez-vous” technique may be more efficient and safer, but it is not straightforward to perform this operation with a single, small-caliber catheter like the 6-Fr guiding catheter. Therefore, the RG3 wire externalization technique was adopted in this case. When the RG3 wire was externalized, the safest method was to pull back the microcatheter up to the distal part of the CTO but keep covering the septal channel with the microcatheter. A new microcatheter was used antegradely and advanced beyond the CTO segment. However, because the 6-FrJL3.5 guiding catheter that was used could not accommodate two microcatheters at the same time, the microcatheter was withdrawn and the same one was used to advance antegradely. Indeed, this procedure risked damaging the uncovered septal collateral channel. To protect the septal collateral, the microcatheter was advanced to the proximal LAD through the septal collateral over the wire antegradely when we removed the RG3 wire.

## Conclusion

This rare case report demonstrated that transradial approach of retrograde PCI for the CTO *via* an ipsilateral septal collateral using a single guiding catheter is feasible and safe in appropriately selected cases.

## Data Availability Statement

The original contributions presented in the study are included in the article/supplementary material, further inquiries can be directed to the corresponding author/s.

## Ethics Statement

Written informed consent was obtained from the individual(s) for the publication of any potentially identifiable images or data included in this article.

## Author Contributions

XL wrote the first draft of the manuscript. JZ wrote sections of the manuscript. HZ and PZ performed the PCI, collected cardiological data, and prepared PCI pictures. NF contributed to the case diagnosis, therapy, and decision-making. All authors contributed to manuscript revision, read, and approved the submitted version.

## Conflict of Interest

The authors declare that the research was conducted in the absence of any commercial or financial relationships that could be construed as a potential conflict of interest.

## Publisher's Note

All claims expressed in this article are solely those of the authors and do not necessarily represent those of their affiliated organizations, or those of the publisher, the editors and the reviewers. Any product that may be evaluated in this article, or claim that may be made by its manufacturer, is not guaranteed or endorsed by the publisher.

## References

[1] Sianos G Barlis P Di Mario C Papafaklis MI Büttner J Galassi AR. European experience with the retrograde approach for the recanalisation of coronary artery chronic total occlusions. A report on behalf of the euro CTO club. Eurointervention. (2008) 4:84–92. 10.4244/EIJV4I1A15 19112784

[2] Kimura M Katoh O Tsuchikane E Nasu K Kinoshita Y Ehara M. The efficacy of a bilateral approach for treating lesions with chronic total occlusions the CART (controlled antegrade and retrograde subintimal tracking) registry. JACC Cardiovasc Interv. (2009) 2:1135–41. 10.1016/j.jcin.2009.09.008 19926057

[3] Zhang B Wong A. The confluent balloon technique for retrograde therapy of chronic total occlusion. Catheter Cardiovasc Interv. (2011) 78:60–4. 10.1002/ccd.22857 21413117

[4] Egred M. Transradial retrograde recanalization of totally occluded left anterior descending artery using a single 7 fr guiding catheter. J Invasive Cardiol. (2013) 25:57–60. 23293178

[5] Brilakis ES Grantham JA Thompson CA DeMartini TJ Prasad A Sandhu GS. The retrograde approach to coronary artery chronic total occlusions: a practical approach. Catheter Cardiovasc Interv. (2012) 79:3–19. 10.1002/ccd.23004 22215566

[6] Rathore S Katoh O Matsuo H Terashima M Tanaka N Kinoshita Y. Retrograde percutaneous recanalization of chronic total occlusion of the coronary arteries: procedural outcomes and predictors of success in contemporary practice. Circ Cardiovasc Interv. (2009) 2:124–32. 10.1161/CIRCINTERVENTIONS.108.838862 20031705

[7] Chon MK Kim JS Chun KJ. Retrograde percutaneous coronary intervention for left anterior descending chronic total occlusion via an ipsilateral intraseptal collateral channel using a single guiding catheter. J Coll Physicians Surg Pak. (2016) 26:S4–6. 27376218

[8] Surmely JF Katoh O Tsuchikane E Nasu K Suzuki T. Coronary septal collaterals as an access for the retrograde approach in the percutaneous treatment of coronary chronic total occlusions. Catheter Cardiovasc Interv. (2007) 69:826–32. 10.1002/ccd.20816 17253598

[9] Thompson CA Jayne JE Robb JF Friedman BJ Kaplan AV Hettleman BD. Retrograde techniques and the impact of operator volume on percutaneous intervention for coronary chronic total occlusions an early U.S. experience. JACC Cardiovasc Interv. (2009) 2:834–42. 10.1016/j.jcin.2009.05.022 19778771

